# Confronting implicit bias toward patients: a scoping review of post-graduate physician curricula

**DOI:** 10.1186/s12909-022-03720-0

**Published:** 2022-09-29

**Authors:** S. T. Gleicher, M. A. Chalmiers, B. Aiyanyor, R. Jain, N. Kotha, K. Scott, R. S. Song, J. Tram, C. L. Vuong, J. Kesselheim

**Affiliations:** 1 Neurology, University of Washington, Seattle, USA; 2 School of Medicine, University of California San Diego, San Diego, USA; 3 Pediatric Emergency Medicine, Children’s Hospital of Philadelphia, Philadelphia, USA; 4 Pediatric Hematology/Oncology, University of California San Francisco, San Francisco, USA; 5 Neonatal Intensive Care, Children’s Hospital of Philadelphia, Philadelphia, USA; 6 Emory University, Atlanta, USA; 7 Emergency Medicine, Advocate Christ Medical Center, Oak Lawn, USA; 8 DFCI/BCH Cancer and Blood Disorders Center, Harvard Medical School, Boston, USA

**Keywords:** Implicit bias, Post graduate medical education, Stereotype, Curriculum, Prejudice

## Abstract

**Background:**

Physicians’ behavior may unknowingly be impacted by prejudice and thereby contribute to healthcare inequities. Despite increasingly robust data demonstrating physician implicit bias (The Office of Minority Health. Minority Population Profiles, 2021; COVID-19 Shines Light on Health Disparities, National Conference of State Legislatures 2021), the evidence behind how to change this with training programs remains unclear. This scoping review therefore reports on the implementation, outcomes, and characteristics of post-graduate physician implicit bias curricula.

**Methods:**

The authors conducted a literature review using scoping review methodology. They searched 7 databases in February and November 2020 for English-language academic and gray literature on implicit bias curricula for physicians at all levels of post-graduate training. Ten reviewers screened studies for eligibility independently, then extracted data from these studies and compiled it into a chart and analytical summary.

**Results:**

Of the 4,599 articles screened, this review identified 90 articles on implicit bias interventions for post-graduate physicians. Inductive data analysis revealed a spectrum of educational approaches, which were categorized int o 4 educational models called Competence, Skills-Based, Social Contact, and Critical Models. The most commonly reported strength was the interactive nature of the curricula (26%), and the most frequently identified challenges were related to time and resources available (53%). Half of the interventions discussed facilitator preparation, and the majority (62%) evaluated outcomes using pre and post self-assessments.

**Conclusions:**

This review provides a comprehensive synthesis of the literature on physician implicit bias curricula. It is our goal that this supports medical educators in applying and improving aspects of these interventions in their own programs.

**Supplementary Information:**

The online version contains supplementary material available at 10.1186/s12909-022-03720-0.

## Background

Longstanding health inequities based on race, gender, socioeconomic status, and other social influencers of health have been the subject of renewed attention in light of current events such as the COVID-19 pandemic and our national reckoning with systemic racism [1, 2]. The Agency for Healthcare Research and Quality reports that patients of Black, LatinX, or indigenous race receive worse care in relation to 40% of quality measures assessed, and the annual National Healthcare Disparities Report consistently demonstrates that white patients receive better quality of care than other racial groups [3]. This differential in care remains after controlling for economic status, educational level, and healthcare access, suggesting discrimination on the part of the medical system [4], where covert prejudice remains present at the individual and institutional levels.

There are numerous factors which contribute to health inequities, but mounting research suggests that implicit bias toward patients may have measurable impacts on healthcare [5]. Implicit bias is an unconscious and unintentional association between a category of people and some attribute [6]. While *explicit* attitudes are deliberate and conscious, *implicit*attitudes can affect behavior without conscious volition [3].

Post-graduate physicians may be an attractive target audience for educational interventions about implicit bias because they are responsible both for making clinical decisions and training future generations of physicians. Despite this, the availability of opportunities for physicians to explore their biases in a formal setting after medical school is unclear, and no literature review has been conducted on post-graduate physician implicit bias interventions [7–9].

We conducted a comprehensive scoping review to present the content and outcomes of educational interventions which address post-graduate physicians’ implicit bias toward patients, to potentially inform decision-making of medical educators seeking similar interventions.

## Methods

We employed a rigorous scoping review methodology, using the JBI Manual for Evidence Synthesis framework [10]. Using this strategy, our scoping review was split into the stages below:

### Developing the research question

We asked, how can implicit bias toward patients be addressed through physician educational programs?

### Inclusion criteria

The population of focus was post-graduate physicians, such as resident physicians, fellows, and attending physicians of all specialties, including populations in which physicians were a subgroup of a larger group of learners. We focused on curricula addressing implicit bias toward patients and defined implicit bias as stigma, prejudice, stereotype, and other forms of unconscious bias based on race, socioeconomic status, sexual orientation, weight, substance use, and any other personal identifying trait. We defined curricula as any planned educational experiences, including clinical rotations, didactics, training programs, and conferences. Primary research, systematic reviews, books, editorials, guidelines, videos, and conference abstracts were included, while non-English language studies were excluded. We included literature describing implemented curricula as well as literature which provided recommendations and theoretical background for potential interventions. We did not limit studies by publication date.

### Search strategy

With the aid of an experienced research librarian (P. Bain, Countway), we conducted a search of MEDLINE (Ovid), Embase, Web of Science, ERIC, CINAHL, and PsycINFO in February 2020 using the search strategy and keywords in Additional file 1: Appendix 1. Because we found relevant articles from the database MedEdPORTAL which were not identified in this initial search, we conducted a manual search of MedEdPORTAL in November 2020 using the terms “implicit bias,” “unconscious bias,” “prejudice,” and “stigma.”

### Sources of evidence selection

**Fig. 1:**
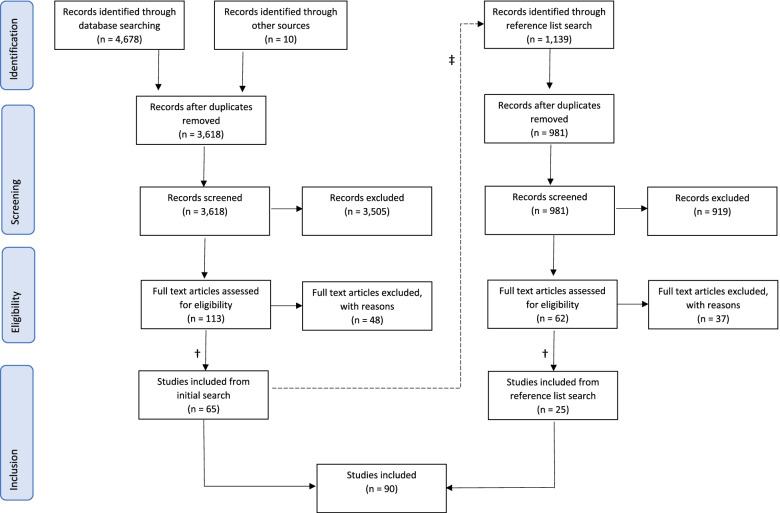
Flowchart of the screening process using PRISMA (Preferred Reporting Items for Systematic Reviews and Meta-Analyses) guidelines [11] † We assigned reviewers for full text review such that the screeners for each article’s full text were different from the screeners for its title/abstract review. This ensured that each article was screened in total by 4 different reviewers, in order to minimize effects of individual biases or subjective interpretations of criteria ‡ We imported sources cited in the bibliographies of included studies into Covidence and repeated the two-phase screening process

We used Covidence systematic review management software (Melbourne, Australia) for each step of screening and data extraction. First, all reviewers applied inclusion criteria to 10% of the papers to ensure that we were uniform in our screening. We conducted the remaining screening in two stages: titles and abstracts were screened initially, then the full texts of included articles were screened to determine final eligibility (Fig. 1). All coauthors (S.G., M.C., B.A., R.J., N.K., K.S., R.S., J.T., C.V., and J.K.) participated in both rounds of screening. Each article was independently reviewed by two coauthors using predefined selection criteria and we resolved disagreements with reviewer discussion until consensus was reached.

### Data extraction

Coauthors (S.G., M.C., B.A., R.J., N.K., K.S., R.S., J.T., C.V., and J.K.) collected data from the included studies using a data extraction form (Additional file 2: Appendix 2). The form’s data fields were guided by educational principles deemed most relevant by the coauthors as well as the Guideline for Reporting Evidence-Based Practice Education Interventions and Teaching (GREET) checklist [12].

### Analysis of the evidence

Three coauthors (S.G., M.C., and J.K.) analyzed data qualitatively and quantitatively, using frequency counts for key characteristics identified. Interventions were categorized into 4 distinct educational models developed iteratively via inductive coding by the authors. We analyzed outcomes using Kirkpatrick’s 4 levels of program evaluation, an analytic model for curricular outcome measurement [13].

## Results

### Curriculum characteristics

Our review identified 90 articles on implicit bias interventions for post-graduate physicians. Table 1 presents the aggregated data from these articles, and Additional file 3: Appendix 3 summarizes characteristics of all 90 articles.

**Table 1:** Aggregated data from 90 studies included in scoping review on post-graduate physician implicit bias curricula

**Type of bias addressed**	n (%)Total reported: 90
General implicit bias	41 (46%)
Race, ethnicity, and diverse cultures	21 (23%)
LGBQ Patients	7 (8%)
Mental Illness	6 (7%)
Socioeconomic Status	6 (7%)
Other Including bias related to HIV/AIDS, weight/obesity, gender, substance use disorders, disability, age, gender non-conforming/intersex, and incarcerated populations	20 (22%)
**Learners' professional position**	n (%) Total reported: 82
Residents/fellows	53 (65%)
Attendings	26 (32%)
Physicians: unspecified	20 (24%)
Mixed health professionals Nurses, social workers, and other members of the health care system	18 (22%)
Medical students	13 (16%)
**Learners' specialty**	n (%) Total reported: 49
Internal medicine Including general internal medicine, hematology-oncology, endocrinology, and primary care	17 (35%)
Family medicine	9 (18%)
Emergency medicine	8 (16%)
Pediatrics	8 (16%)
Open to multiple specialties	4 (8%)
Psychiatry	4 (8%)
Other Including OB/GYN, physical medicine and rehabilitation, surgery, and palliative care	5 (10%)
**Curriculum schedule**	n (%) Total reported: 52
Single session	28 (54%)
6 months or more	11 (21%)
1 month to < 6 months	8 (15%)
1 week to < 4 weeks	4 (8%)
2 days to < 7 days	1 (2%)
**Mode of intervention**	n (%) Total reported: 73
Group discussion, exercise, or debrief	49 (67%)
Lecture, didactic, or reading	41 (56%)
Exposure to patient population or community members	20 (27%)
Reflection exercise or writing	16 (22%)
Film	15 (21%)
Role play or simulation	13 (18%)
IAT	11 (15%)
Case-based learning	10 (14%)
Asynchronous online module or e-learning	5 (7%)
**Was facilitator background/preparation reported?**	n (%) 66 implemented curriculum
Yes	33 (50%)
No	33 (50%)
**Methods for measuring outcomes**	n (%) Total reported: 58
Pre and post surveys	36 (62%)
Post surveys/course evaluations	19 (33%)
Interviews/focus groups	8 (14%)
Observation of clinical decision-making	3 (5%)
Long-term follow-up surveys	3 (5%)
Other Includes written reflections and IAT	3 (5%)
**Outcomes reported**	n (%) Total reported: 53
Increased recognition of systemic disparities	19 (36%)
Increased awareness of personal bias	15 (28%)
Significant reduction in measured bias	15 (28%)
Increased comfort in or commitment to addressing bias	14 (26%)
Learners rated intervention highly	8 (15%)
Self-reported reduction in discriminatory behavior	7 (13%)
Increased knowledge of strategies to address bias	7 (13%)
Increased understanding of patients' experiences	4 (8%)
Increased insight into teaching about bias	3 (6%)
Other: Includes significant increase in measured bias and no significant change in learner behavior	2 (4%)
**Strengths reported**	n (%) Total reported: 35
Group discussion/interactive	9 (26%)
Self-reflection on personal bias	7 (20%)
Demonstrates heterogeneity within stereotyped groups (by breaking down ingroup/outgroup boundaries or through exposure to stereotyped groups)	7 (20%)
Evidence-based Research or guidelines formed basis for curriculum	6 (17%)
Perspective-taking/fosters empathy	5 (14%)
Interdisciplinary contributions to curriculum Involving patients, community, or other fields	5 (14%)
Learning environment conducive to honest discussion	5 (14%)
Cultural humility/cross-cultural care	5 (14%)
Feasibility	4 (11%)
Actionable solutions Provides tools for providers to use to change clinical practice	4 (11%)
Simulated patient encounter	3 (9%)
**Weaknesses**	n (%) Total reported: 36
Lack of time/resources Includes scheduling challenges, brief duration of intervention, and lack of faculty/institutional investment	19 (53%)
Learner defensiveness (including distrust of IAT validity)	7(19%)
Lack of facilitators experienced in/comfortable with subject material	5 (14%)
Learners self-selected and may not represent target audience	4 (11%)
Lack of actionable solutions	4 (11%)
Limited scope of course material	3 (8%)
Subject undervalued by learners	3 (8%)
Risk of reinforcing stereotypes	2 (6%)
**Future directions**	n (%) Total reported: 45
Improve outcomes evaluation (including behavioral outcomes and long-term outcomes)	19 (42%)
Extend to more sessions	7 (16%)
Improve facilitator preparation	4 (9%)
Encourage institutional buy-in	3 (7%)
Interdisciplinary and community collaboration Includes partnerships with community, patients, and other disciplines	3 (7%)
Reevaluate competency model Examine alternatives to the cultural competency model for teaching implicit bias	3 (7%)
More clinical immersion	3 (7%)

### Educational models

Inductive data analysis revealed 4 educational models used in implicit bias curricula: Competence, Skills-Based, Social Contact, and Critical Models. Their different theoretical foundations and pedagogical approaches are summarized in Table 2.

**Table 2:** Educational models identified in curricula addressing post-graduate physicians’ implicit bias toward patients

Educational model	Description	n (%) Total reported:
Competence Models	Seek to increase learners’ knowledge about diverse populations and awareness of their own implicit bias, often via self-reflection exercises. Often informed by Pedersen’s [14] foundational Awareness/Knowledge/Skills prototype for culture-centered counseling	30 (54%)
Critical Models	Contextualize implicit bias within larger systems of inequity and seek to prepare learners to catalyze structural change that extends beyond individual clinical interactions	11 (20%)
Skills-Based Models	Employ self-reflection combined with training in specific, evidence-based strategies from Social Cognitive Psychology (e.g. individuation, perspective-taking)	9 (17%)
Social Contact Models	Incorporate evidence from Social Cognitive Psychology to facilitate interactions between clinicians and diverse patients under conditions [15] intended to reduce bias	6 (11%)

### Outcomes reported

**Fig. 2:**
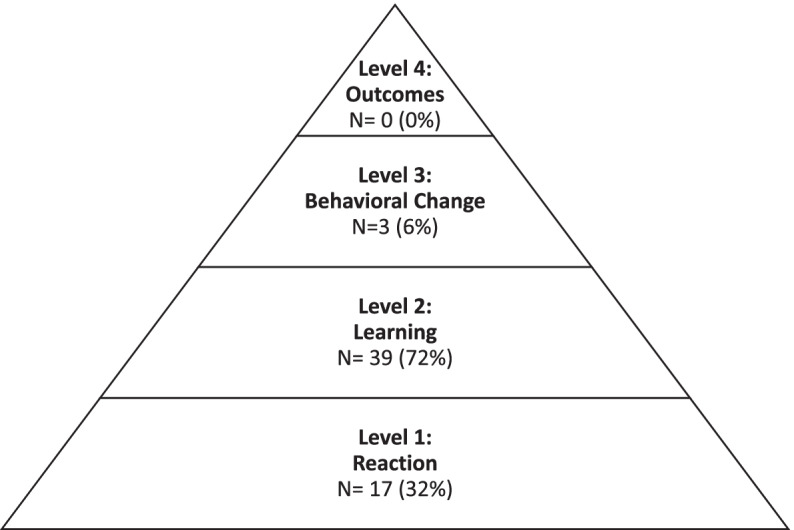
Number (%) falling into each of the 4 levels of Kirkpatrick’s Triangle for Program Evaluation [13], of 53 articles identified

Eighty percent of the educational interventions reported outcomes. Outcome assessments most frequently relied on learners to self-report the perceived effects of the curriculum through pre and post surveys (62%). Figure 2 depicts the interventions’ approaches to outcome measurement through the lens of Kirkpatrick’s model for program evaluation [13].

### Curriculum analysis

Most of the educational methods employed were interactive (67%), and this was the most commonly identified curricular strength (26%). The most common weaknesses identified were related to resource availability, such as schedule and timing, funding, and institutional investment (53%).

## Discussion

Our review identified several elements and challenges of effective physician implicit bias curricula. Below we highlight a spectrum of educational approaches to these curricula, as well as areas for improvement in implementation and outcome assessment.

### Educational models

The 4 educational models (Table 2) identified in our analysis present various strengths and weaknesses. Competence Models have been critiqued for presenting implicit bias as a problem to be understood and resolved at the level of the individual [15–17], often by increasing learners’ awareness of their bias. Although evidence does not support the premise that increased awareness alone will allow clinicians to manage their own implicit bias [18, 19], self-reflection may trigger cognitive dissonance and increase learner motivation to change. In our review, 20% of interventions identified self-reflection on personal bias as a strength. On the other hand, when Competence Models are used to improve learners’ understanding of cultural groups by focusing on categorical traits rather than individuation, they may have the counterproductive effect of actually increasing reliance on stereotypes [20–22]. It is critical that interventions demonstrate heterogeneity rather than homogeneity within stereotyped groups, a strength which was recognized in 20% of curricula published in this review.

Skills-Based Models draw upon evidence-based strategies in Social Cognitive Psychology that aim to reduce stereotyping outside of healthcare settings [18, 23–25]. These skills may include “perspective-taking,” which fosters empathy by asking learners to imagine themselves in a patient’s position. Another practice, called individuation, consciously focuses on “specific information about an individual,” [18] which may “increase [learners’] capacity to see others as members of a common ingroup” instead of an outgroup [23]. Such models sometimes employ mindfulness, which encourages “attention to one’s own thought processes…and how they affect decisions so that one pays attention to the details of clinical care rather than falling back on habits…such as stereotypes” [20].

Social Contact Models facilitate direct interaction with diverse patients to foster empathy and enhance learners’ comfort, confidence, and positive emotions in interactions with people they perceive to be outgroup members [23, 24, 26, 27]. Evidence suggests that social contact only leads to these positive outcomes in specific conditions, namely, the presence of shared goals and equal status between both parties [20, 27]. Otherwise, such interactions have the potential to strengthen previously held stereotypes [20, 27]. To address this risk, novel approaches incorporate standardized patient encounters with debriefing [20]. One downside to Social Contact Models is that lessons learned with specific populations may not be easily applied to other contexts, in contrast to Skills-Based Models, which provide tools meant to be universally applicable.

Critical Models seek to profoundly transform the paradigms through which learners think about equity and justice in the medical system. In contrast to other models, which seek to avoid provoking discomfort or defensiveness among learners [16, 20], Critical Models intentionally present learners with experiences designed to arouse emotions, destabilize assumptions, and trigger cognitive dissonance. According to transformative learning [19, 28, 29], an educational theory which focuses on adult learning, such an exposure to a “disorienting dilemma” [30] prompts learners to “engage in a process of self-examination,” leading to paradigm shift [31].

### Curriculum implementation

Each educational model encountered challenges in its implementation. Our review revealed barriers related to institutional investment and culture, availability of experienced facilitators, and learner-related factors.

Institutional attitudes can support or impede learning by impacting the time and funding available for implicit bias programs [29]. Given the multiple competing demands for medical staff time [32], it is unsurprising that over half of the interventions held only a single session, despite concern that “the lessons of a onetime workshop…tend to fade as the volume of work increases, and old practices reassert themselves” [33]. When institutional investment is lacking, the burden is carried by a handful of sometimes overtaxed individuals, as one author recalls, “we had momentum. What we didn’t have was money…which was a recipe for a lot of talk and no action…it seemed pretty clear I was going to have to find the funding for it myself” [34]. We also observed an uneven distribution of implicit bias programs between various specialties, illustrating how departmental subcultures may affect the accessibility of such trainings.

Another barrier identified was the availability of facilitators who were comfortable and well-versed in the subject matter [20, 26, 29, 33, 35, 36]. Only half of the interventions discussed the training of facilitators. A deficiency of experienced facilitators could detract from curriculum feasibility and quality while compounding variability in learner experiences. Facilitators may be wary of teaching implicit bias because of the sensitivity of the subject matter, inadequate preparation and training, or institutional cultures of silence with relation to bias [29]. Some questioned the evidence behind implicit bias, or felt antagonized when confronted with inequities in their establishment [34]. In response, several articles investigated best practices for facilitator training and identified this as a crucial area for future research [15, 29, 37].

Implicit bias programs were also impacted by factors related to learners. Multiple studies relayed concerns that the voluntary nature of these curricula meant that attendees were “self-selected,” [38] such that the program may have been “preaching to the choir.” Interventions can reach a greater array of learners if their institutions value implicit bias training and support learners in making time for it [26]. Changing institutional culture may also address another learner-related factor: the defensiveness and feelings of shame, fear [29] or denial [39] that may be experienced when confronting one’s own bias. Although such discomfort can be part of the process, as in the case of Critical Models [30, 31], too much discomfort can be counterproductive. Educators should provide a supportive environment to intentionally channel learner discomfort into behavioral change [20, 31].

Environments which support vulnerability and are free of criticism are optimal if learners are to experience transformative change [16]. One study suggested that “self-reflection, self-awareness, discovering…of often shameful past experiences of bias—could only be accomplished through…a non-judgmental environment in which everyone feels comfortable expressing their views with little fear of mockery or embarrassment” [16]. It is also crucial to avoid taxing learners who are underrepresented minorities by treating them as token representatives of their group or expecting them to educate other learners [40]. Educators must strive to “create a learning environment that fosters safety, trust, and respect,” “vet speakers, content, and materials carefully,” and “employ andragogical versus pedagogical methods of learning” which treat learners as active agents in their own learning [41]. Striking this balance may be especially difficult when power differentials exist between facilitators and trainees, which reinforces the need for robust faculty development [29].

### Outcomes reported

Program evaluation is an essential component of curriculum development [42, 43]. Seventeen percent of studies in this review labeled evidence supporting interventions as a strength. This suggests that educators are seeking data to guide curricula, yet 20% of interventions did not report results. Faculty development initiatives should explicitly encourage educators to create a prospective evaluation plan to measure and disseminate outcomes, so that others may benefit from the lessons learned.

Kirkpatrick’s model for program evaluation (Fig. 2) is a well-known paradigm to categorize approaches to outcome measurement. The reported outcomes of included publications most commonly mapped to Level 2: Learning, which relates to learners’ knowledge, attitudes, or skills, as well as confidence or commitment to change [13]. Noteworthy shortcomings exist within this subset of data. While optimal measurement at Level 2 would involve an external evaluator [42], many studies reported outcomes via self-assessments, raising concerns about their validity [44]. As an alternative, several authors measured IAT scores, often in a pre/post intervention format. The advantages of such an approach are the rigor with which IAT instruments are developed and evidence that the IAT has greater predictive validity than other self-report measures [45], but some publications question the validity and precision of IAT-based data [46–48].

Few included studies attempted to measure outcomes at Kirkpatrick Levels 3–4. Level 3 assesses the degree to which learners apply what they learned, and Level 4 assesses targeted outcomes and organizational benefits [13]. Although measurement at these higher levels is challenging due to the time, money, and methodologic expertise required [49, 50], investing in such outcome evaluation presents the best opportunity to demonstrate meaningful impact on physician implicit bias and patient care [13]. Many of the interventions described in this review do not measure efficacy at these higher level outcomes, a limitation which has been recognized in prior implicit bias research [51]. Educators wishing to adopt similar curricula should understand that evidence directly supporting these interventions’ reduction of implicit bias in the clinical or learning environments is lacking. It is our hope that with higher level outcome assessment, more longitudinal interventions employing engaging teaching modalities, increased faculty training, and organizational culture eager to address implicit bias, our field will refine implicit bias curricula and benefit from more compelling data supporting them.

## Conclusions

Our analysis of the literature on post-graduate physician implicit bias curricula highlights opportunities for next steps in the field:

1. Educators seeking implicit bias curricula can consider the educational models, teaching modalities, and challenges identified in this review to critically apply and improve aspects of these interventions in their own programs.
2. Institutional investment and faculty development were commonly identified challenges in this review of implicit bias curricula. Educators should examine whether their organizational culture, leaders, and teaching faculty will support implicit bias curricula and commit needed resources.
3. Implicit bias curricula should be evidence-based. This requires more widespread program evaluation using well-validated instruments, and especially assessing changes in physician behavior and impacts on patients.

### Limitations

This scoping review presents an extensive yet incomplete snapshot of implicit bias curricula for physicians. It is limited to the databases we searched, although we identified additional papers through the iterative process of screening included studies’ bibliographies. In addition, many articles provided only brief information in the form of an abstract. Each stage of screening, data extraction, and coding likely introduced a degree of bias from the reviewers, which we mitigated by having 2 reviewers reach consensus at each step. Finally, per scoping review methodology, we did not consider the quality of the studies we included. This lack of discrimination should be considered when extrapolating results.

## Supplementary Information

**Additional file 1: Appendix 1.** Search strategy of post-graduate physician implicit bias curricula in MEDLINE (Ovid), Embase, Web of Science, ERIC, CINAHL, and PsycINFO in February 2020.

**Additional file 2: Appendix2.** Data extraction form for scoping review on curricula addressing post-graduate physician implicit bias toward patients.

**Additional file 3: Appendix 3.** Summary of all 90 studies included in the scoping review (February-November 2020) of post-graduate physician implicit bias curricula [52–119].

## Data Availability

The datasets supporting the conclusions of this article are available in the following repositories: MEDLINE (Ovid), https://www.wolterskluwer.com/en/solutions/ovid/ovid-medline-901 Embase, https://www.embase.com/ Web of Science, https://www.webofscience.com ERIC, https://eric.ed.gov CINAHL, https://www.ebsco.com/products/research-databases/cinahl-database PsycINFO, https://www.apa.org/pubs/databases/psycinfo MedEdPORTAL, https://www.mededportal.org

## References

[1] The Office of Minority Health. Minority Population Profiles. https://minorityhealth.hhs.gov/omh/browse.aspx?lvl=2&lvlid=26. Accessed 17 June 2021.

[2] COVID-19 Shines Light on Health Disparities > National Conference of State Legislatures. https://www.ncsl.org/blog/2020/07/30/covid-19-shines-light-on-health-disparities.aspx. Accessed 17 June 2021.

[3] Hall WJ, Chapman MV, Lee KM (2015). Implicit racial/ethnic bias among health care professionals and its influence on health care outcomes: a systematic review. Am J Public Health.

[4] Zestcott CA, Blair IV, Stone J (2016). Examining the presence, consequences, and reduction of implicit bias in health care: a narrative review. Group Process Intergroup Relat GPIR.

[5] Dehon E, Weiss N, Jones J, Faulconer W, Hinton E, Sterling S (2017). A Systematic review of the impact of physician implicit racial bias on clinical decision making. Acad Emerg Med Off J Soc Acad Emerg Med.

[6] Gonzalez CM, Deno ML, Kintzer E, Marantz PR, Lypson ML, McKee MD (2018). Patient perspectives on racial and ethnic implicit bias in clinical encounters: Implications for curriculum development. Patient Educ Couns.

[7] Morris M, Cooper RL, Ramesh A (2019). Training to reduce LGBTQ-related bias among medical, nursing, and dental students and providers: a systematic review. BMC Med Educ.

[8] Sukhera J, Wodzinski M, Rehman M, Gonzalez CM (2019). The implicit association test in health professions education: a meta-narrative review. Perspect Med Educ.

[9] Brottman MR, Char DM, Hattori RA, Heeb R, Taff SD (2020). Toward cultural competency in health care: a scoping review of the diversity and inclusion education literature. Acad Med J Assoc Am Med Coll.

[10] Peters M, Godfrey C, McInerney P, Munn Z, Trico A, Khalil H. Chapter 11: Scoping Reviews. In: Aromataris E, Munn Z, eds. JBI Manual for Evidence Synthesis. JBI; 2020 10.46658/JBIMES-20-12

[11] Moher D, Liberati A, Tetzlaff J, Altman DG (2009). Preferred reporting items for systematic reviews and meta-analyses: the PRISMA statement. BMJ.

[12] Phillips AC, Lewis LK, McEvoy MP (2016). Development and validation of the guideline for reporting evidence-based practice educational interventions and teaching (GREET). BMC Med Educ.

[13] Kirkpatrick D, Kirkpatrick J. Evaluating Training Programs. 3rd ed. Berrett-Koehler Publishers; 2006.

[14] Position Paper: Cross-Cultural Counseling Competencies - Derald Wing Sue, Joseph E. Bernier, Anna Durran, Lawrence Feinberg, Paul Pedersen, Elsie J. Smith, Ena Vasquez-Nuttall, 1982. https://journals.sagepub.com/doi/10.1177/0011000082102008. Accessed 19 May 2021.

[15] White-Davis T, Edgoose J, Speights JB, et al. Addressing Racism in Medical Education An Interactive Training Module. Fam Med. 2018;50(5):364–8. 10.22454/FamMed.2018.875510 29762795

[16] Hannah SD, Carpenter-Song E (2013). Patrolling your blind spots: introspection and public catharsis in a medical school faculty development course to reduce unconscious bias in medicine. Cult Med Psychiatry.

[17] Metzl JM, Hansen H. Structural competency: theorizing a new medical engagement with stigma and inequality. Soc Sci Med. 2014;103:126–33. 10.1016/j.socscimed.2013.06.032. 10.1016/j.socscimed.2013.06.032 PMC4269606 24507917

[18] Chapman EN, Kaatz A, Carnes M (2013). Physicians and implicit bias: how doctors may unwittingly perpetuate health care disparities. J Gen Intern Med.

[19] Sherman MD, Ricco J, Nelson SC, Nezhad SJ, Prasad S (2019). Implicit bias training in a residency program: aiming for enduring effects. Fam Med.

[20] Teal CR, Gill AC, Green AR, Crandall S (2012). Helping medical learners recognise and manage unconscious bias toward certain patient groups. Med Educ.

[21] Paroz S, Bonvin R, Casillas A (2014). Cultural competence education in a simulated clinical environment: a pilot experience. J Gen Intern Med.

[22] Razack S (2007). Promoting skill-building in cultural competence: a must for paediatricians who care for socially vulnerable populations. Paediatr Child Health.

[23] Burgess D, van Ryn M, Dovidio J, Saha S (2007). Reducing racial bias among health care providers: lessons from social-cognitive psychology. J Gen Intern Med.

[24] Knaak S, Mantler E, Szeto A (2017). Mental illness-related stigma in healthcare: Barriers to access and care and evidence-based solutions. Healthc Manage Forum.

[25] Perdomo Joanna, Tolliver Destiny, Hsu Heather, et al. Health Equity Rounds: An Interdisciplinary Case Conference to Address Implicit Bias and Structural Racism for Faculty and Trainees. MedEdPORTAL. 15:10858 10.15766/mep_2374-8265.10858. 10.15766/mep_2374-8265.10858 PMC7050660 32166114

[26] Agrawal S, Capponi P, López J (2016). From surviving to advising: a novel course pairing mental health and addictions service users as advisors to senior psychiatry residents. Acad Psychiatry.

[27] Sukhera J, Miller K, Scerbo C, Milne A, Lim R, Watling C. Implicit Stigma Recognition and Management for Health Professionals. Acad Psychiatry. 2020;44(1):59-63. 10.1007/s40596-019-01133-8. 10.1007/s40596-019-01133-8 31701387

[28] Loignon C, Boudreault-Fournier A, Truchon K, Labrousse Y, Fortin B (2014). Medical residents reflect on their prejudices toward poverty: a photovoice training project. BMC Med Educ.

[29] Gonzalez CM, Garba RJ, Liguori A, Marantz PR, Diane McKee M, Lypson ML (2018). How to make or break implicit bias instruction: implications for curriculum development. Acad Med J Assoc Am Med Coll.

[30] Mezirow J (1997). Transformative learning: theory to practice. New Dir Adult Contin Educ.

[31] Sukhera J, Watling CJ, Gonzalez CM (2020). Implicit bias in health professions: from recognition to transformation. Acad Med J Assoc Am Med Coll.

[32] Ingraham N, Magrini D, Brooks J, Harbatkin D, Radix A, Haynes SG (2016). Two tailored provider curricula promoting healthy weight in lesbian and bisexual women. Womens Health Issues.

[33] Cahn Peter S. Recognizing and Reckoning With Unconscious Bias: A Workshop for Health Professions Faculty Search Committees. MedEdPORTAL. 2017;13:10544. 10.15766/mep_2374-8265.10544. 10.15766/mep_2374-8265.10544 PMC6354718 30800746

[34] Seeing Patients — Augustus A. White III, MD | Harvard University Press. https://www.hup.harvard.edu/catalog.php?isbn=9780674049055. Accessed 12 Jan 2021.

[35] Zeidan AJ, Khatri UG, Aysola J, et al. Implicit bias education and emergency medicine training: step one? Awareness. Aem Educ Train. 2019;3(1):81–5. 10.1002/aet2.10124. 10.1002/aet2.10124 PMC6339553 30680351

[36] Neely KL, Stifel EN, Milberg LC (2000). A systematic approach to faculty development in Women’s health: lessons from education, feminism, and conflict theory. Acad Med.

[37] Acosta D, Ackerman-Barger K (2017). Breaking the silence: time to talk about race and racism. Acad Med.

[38] Adelekun AA, Beltrán S, Carney J (2019). Recognizing racism in medicine: a student-organized and community-engaged health professional conference. Health Equity.

[39] Holm AL, Rowe Gorosh M, Brady M, White-Perkins D (2017). Recognizing privilege and bias: an interactive exercise to expand health care providers’ personal awareness. Acad Med.

[40] Rodríguez JE, Campbell KM, Pololi LH (2015). Addressing disparities in academic medicine: what of the minority tax?. BMC Med Educ.

[41] Like RC (2011). Educating clinicians about cultural competence and disparities in health and health care. J Contin Educ Health Prof.

[42] Frye AW, Hemmer PA (2012). Program evaluation models and related theories: AMEE Guide No. 67. Med Teach..

[43] Thomas PA, Kern DE, Hughes MT, Chen BY. Curriculum Development for Medical Education: A Six-Step Approach. Johns Hopkins University Press; 2015. 10.1353/book.44600.

[44] Eva K, Regehr G (2008). “I’ll never play professional football” and other fallacies of self-assessment. J Contin Educ Health Prof.

[45] Greenwald AG, Poehlman TA, Uhlmann EL, Banaji MR (2009). Understanding and using the Implicit Association Test: III. Meta-analysis of predictive validity. J Pers Soc Psychol..

[46] Olson MA, Fazio RH (2003). Relations between implicit measures of prejudice:what are we measuring?. Psychol Sci.

[47] Olson MA, Fazio RH (2004). Reducing the influence of extrapersonal associations on the Implicit association test: personalizing the IAT. J Pers Soc Psychol.

[48] Han HA, Czellar S, Olson MA, Fazio RH (2010). Malleability of attitudes or malleability of the IAT?. J Exp Soc Psychol.

[49] Kirkpatrick DL (2006). Seven keys to unlock the four levels of evaluation. Perform Improv.

[50] Kennedy PE, Chyung SY, Winiecki DJ, Brinkerhoff RO (2014). Training professionals’ usage and understanding of Kirkpatrick’s Level 3 and Level 4 evaluations. Int J Train Dev.

[51] FitzGerald C, Martin A, Berner D, Hurst S (2019). Interventions designed to reduce implicit prejudices and implicit stereotypes in real world contexts: a systematic review. BMC Psychol.

[52] Pinderhughes E. Understanding Race, Ethnicity, and Power: The Key to Efficacy in Clinical Practice. Simon and Schuster; 1989.

[53] Marr B, Mickey SH, Blythe SG, Baruch J. The Weight of Pain: What Does a 10 on the Pain Scale Mean? An Innovative Use of Art in Medical Education to Enhance Pain Management. J Pain Symptom Manage. 2019;57(6):1182-7. 10.1016/j.jpainsymman.2019.03.016. 10.1016/j.jpainsymman.2019.03.016 30905676

[54] Bayar MR, Poyraz BC, Aksoy-Poyraz C, Arikan MK. Reducing mental illness stigma in mental health professionals using a web-based approach. Isr J Psychiatry Relat Sci. 2009;46(3):226-30. 20039525

[55] Smith WR, Betancourt JR, Wynia MK, et al. Recommendations for teaching about racial and ethnic disparities in health and health care. Ann Intern Med. 2007;147(9):654-655. 10.7326/0003-4819-147-9-200711060-00010. 10.7326/0003-4819-147-9-200711060-00010 17975188

[56] Richardson HB, Guralnick MJ. Pediatric residents and young handicapped children: Curriculum evaluation. J Med Educ. 1978;53(6):487-92. 10.1097/00001888-197806000-00005 149201

[57] Bryce V, Sullivan C, Hall C, Wang W, Ng A. A multidisciplinary and culturally appropriate model of care in cardiac outreach clinic improves indigenous patient continuity of care. Heart Lung Circ. 2012;21:S301-S302. 10.1016/j.hlc.2012.05.744.

[58] Alonzo CA. Uthscsa safe space: Becoming an ally to the lgbtq communities. J Gen Intern Med. 2014;29:S542.

[59] Lim RF, Diamond RJ, Chang JB, Primm AB, Lu FG. Using Non-Feature Films to Teach Diversity, Cultural Competence, and the DSM-IV-TR Outline for Cultural Formulation. Acad Psychiatry. 2008;32(4):291-8. 10.1176/appi.ap.32.4.291 18695030

[60] Dennis SN, Gold RS, Wen FK. Learner Reactions to Activities Exploring Racism as a Social Determinant of Health. Fam Med. 2019;51(1):41-7. 10.22454/FamMed.2019.704337. 10.22454/FamMed.2019.704337 30633797

[61] Moroz A, Gonzalez-Ramos G, Festinger T, Langer K, Zefferino S, Kalet A. Immediate and follow-up effects of a brief disability curriculum on disability knowledge and attitudes of PM&R residents: A comparison group trial. Med Teach. 2010;32(8):e360-e364. 10.3109/0142159X.2010.490602. 10.3109/0142159X.2010.490602 20662571

[62] Rickert CG, Perez NP, Westfal ML, et al. Understanding Our Own Biases as Surgeons: A Departmental Effort. Ann Surg. 2020;271(1):39-40. 10.1097/SLA.0000000000003392. 10.1097/SLA.0000000000003392 31188209

[63] Baig AA, Benitez A, Paredes AZ, et al. Local patients, local stories: A Latino cultural competency training program for healthcare providers. J Gen Intern Med. 2014;29:S140-S141.

[64] Lohiniva AL, Benkirane M, Numair T, et al. HIV stigma intervention in a low-HIV prevalence setting: a pilot study in an Egyptian healthcare facility. AIDS Care. 2016;28(5):644-652. 10.1080/09540121.2015.1124974. 10.1080/09540121.2015.1124974 26717980

[65] Backhus LM, Lui NS, Cooke DT, Bush EL, Enumah Z, Higgins R. Unconscious Bias: Addressing the Hidden Impact on Surgical Education. Thorac Surg Clin. 2019;29(3):259-67. 10.1016/j.thorsurg.2019.03.004. 10.1016/j.thorsurg.2019.03.004 31235294

[66] Okubanjo O, Lovell E. Healthcare disparities. West J Emerg Med. 2017;18:S51.

[67] Siegelman JN, Woods C, Salhi B, Heron S. Health care disparities education using the implicit association test. Med Educ. 2016;50(11):1158-9. 10.1111/medu.13174. 10.1111/medu.13174 27762020

[68] Forbes LJ. Food addiction: An overlooked cause of persistent overweight and obesity. 2014;74. http://ezp-prod1.hul.harvard.edu/login?url=http://search.ebscohost.com/login.aspx?direct=true&db=psyh&AN=2014-99080-553&site=ehost-live&scope=site.

[69] Omori A, Tateno A, Ideno T, et al. Influence of contact with schizophrenia on implicit attitudes towards schizophrenia patients held by clinical residents. BMC Psychiatry. 2012;12. http://ezp-prod1.hul.harvard.edu/login?url=http://search.ebscohost.com/login.aspx?direct=true&db=psyh&AN=2014-48254-001&site=ehost-live&scope=site. 10.1186/1471-244X-12-205 PMC3539926 23173747

[70] Eagleton S, Fugate CS, Merten MJ, Welch GL, Harrist AW. Improving physician self-efficacy and reducing provider bias: A family science approach to pediatric obesity treatment. Fam Resil Chronic Illn Interdiscip Transl Perspect. 2017:91-113. 10.1007/978-3-319-26033-4_5.

[71] Bristol S, Kostelec T, MacDonald R. Improving Emergency Health Care Workers’ Knowledge, Competency, and Attitudes Toward Lesbian, Gay, Bisexual, and Transgender Patients Through Interdisciplinary Cultural Competency Training. J Emerg Nurs. 2018;44(6):632-9. 10.1016/j.jen.2018.03.013. 10.1016/j.jen.2018.03.013 29704979

[72] Yang CJ, Thompson DM, Cabrera-Muffly C, Hinni ML. Implicit bias affects us all: Simulation and panel discussion. Otolaryngol Head Neck Surg. 2019;161(2):P30. 10.1177/0194599819858140.

[73] Maksimowski K, Massarella D, Ghori A, et al. Implementing a self-developed cultural competency workshop in pediatric residency and assessing outcomes. Acad Pediatr. 2016;16(6):e47-e48.

[74] Peralta JB, Smith DF, Duh-Leong C, Durstenfeld A, Acholonu RG. Impact of social determinants of health curriculum on resident empathy. Acad Pediatr. 2018;18(5):e2.

[75] Nelson SC, Prasad S, Hackman HW. Training providers on issues of race and racism improve health care equity. Pediatr Blood Cancer. 2015;62(5):915-7. 10.1002/pbc.25448. 10.1002/pbc.25448 25683782

[76] Dielissen PW, Verdonk P, Bottema BJ, Lagro-Janssen TL. Evaluating the teaching of gender-specific medicine in postgraduate training for general practitioners. J Eval Clin Pract. 2009;15(6):1226-9. 10.1111/j.1365-2753.2009.01183.x. 10.1111/j.1365-2753.2009.01183.x 20367733

[77] Lightfoot A, Chapman M, Colby R, et al. Envisioning health: A trans-disciplinary, community engaged visual intervention for healthcare providers on implicit bias toward Latino/a immigrant youth. J Adolesc Health. 2015;56(2):S91. 10.1016/j.jadohealth.2014.10.182.

[78] Wu D, Saint-Hilaire L, Pineda A, et al. The Efficacy of an Antioppression Curriculum for Health Professionals. Fam Med. 2019;51(1):22-30. 10.22454/FamMed.2018.227415. 10.22454/FamMed.2018.227415 30412265

[79] Rincon-Subtirelu M. Education as a tool to modify anti-obesity bias among Pediatric residents. Int J Med Educ. 2017;8:77-78. 10.5116/ijme.58b1.46e3. 10.5116/ijme.58b1.46e3 PMC5357544 28284176

[80] Sabin J, Van Schaik E, Lynch E, Stoner S. Does awareness of unconscious associations enhance learning about healthcare disparities? Am J Epidemiol. 2010;171:S129. 10.1093/aje/kwq151.

[81] Norlock F, Sadowski L, Kapolnek M. A decade of change in attitudes toward the homeless among primary care internal medicine residents. J Gen Intern Med. 2014;29:S500-S501.

[82] Cropper-Williams D. Culturally appropriate training to build better relationships between men who have sex with men (MSM) of color and their health providers. Sex Transm Dis. 2018;45:S109.

[83] Diaz Del Carpio RO, Lema PC, Makdissi R, Dubocovich ML, Burke BA. Cultural and structural competency training for medical residents. J Gen Intern Med. 2018;33(2):696-7.

[84] Ogilvie J, Sangha J, Bertman K, Gerber J, Chen B. Cultivating compassionate care, advocacy skills and a health equity lens in resident physicians: The development of a social paediatrics curriculum. Paediatr Child Health Can. 2019;24:e60. 10.1093/pch/pxz066.0149.

[85] Katz AM, Conant L Jr, Inui TS, Baron D, Bor D. A council of elders: creating a multi-voiced dialogue in a community of care. Soc Sci Med. 2000;50(6):851-60. 10.1016/s0277-9536(99)00341-x 10695982

[86] Stahr A, Kaatz A, Alexander L, et al. Evaluation of a workshop intervention to reduce racial bias in internal medicine residents’ clinical decision-making. J Gen Intern Med. 2017;32(2):S676-S677.

[87] Seybold D, Calhoun B, Burgess D, Lewis T, Gilbert K, Casto A. Evaluation of a Training to Reduce educe Provider Bias Toward Pregnant Patients With Substance Abuse. J Soc Work Pract Addict. 2014;14(3):239-49. 10.1080/1533256X.2014.933730 PMC4508864 26207103

[88] Ufomata E, Eckstrand KL, Hasley P, Jeong K, Rubio D, Spagnoletti C. Comprehensive Internal Medicine Residency Curriculum on Primary Care of Patients Who Identify as LGBT. Lgbt Health. 2018;5(6):375-380. 10.1089/lgbt.2017.0173. 10.1089/lgbt.2017.0173 30141734

[89] Sanchez S, Aysola J. Am i biased? Using the implicit association test to start the conversation among internal medicine residents. J Gen Intern Med. 2018;33(2):683-4.

[90] Tsai J, Brooks K, DeAndrade S, et al. Addressing racial bias in wards. Adv Med Educ Pract. 2018;9:691-6. 10.2147/amep.S159076. 10.2147/AMEP.S159076 PMC6165722 30310343

[91] Pereda B, Montoya M. Addressing Implicit Bias to Improve Cross-cultural Care. Clin Obstet Gynecol. 2018;61(1):2-9. 10.1097/GRF.0000000000000341. 10.1097/GRF.0000000000000341 29300198

[92] Klein EW, Nakhai M. Caring for LGBTQ patients: Methods for improving physician cultural competence. Int J Psychiatry Med. 2016;51(4):315-24. 10.1177/0091217416659268. 10.1177/0091217416659268 27497452

[93] Shutak CW. An academic half-day for healthcare disparities and social justice. Acad Pediatr. 2017;17(5):e20. 10.1016/j.acap.2017.04.072.

[94] Pryce PA, Uwemedimo O, Goenka P, Barone S. 86. EARLY IMPACT OF A HEALTH EQUITY, DIVERSITY, AND INCLUSION CURRICULA ON RESIDENT KNOWLEDGE, ATTITUDES AND SKILL IN CROSS-CULTURAL. CARE. Acad Pediatr. 2019;19(6):e39. 10.1016/j.acap.2019.05.100.

[95] Daetwyler Christof, Schindler Barbara, Parran Ted. The Clinical Assessment of Substance Use Disorders. MedEdPORTAL. 2012;8. 10.15766/mep_2374-8265.9110.

[96] Lypson Monica, Ross Paula, Joiner Terence, Kumagai Arno. Using Multimedia in Faculty Development on Multicultural Education: Scenes From the Movie “Crash.” MedEdPORTAL. 2010;6. 10.15766/mep_2374-8265.8008.

[97] Nageswara Rao A, Warad D, Rodriguez V. Cross-Cultural Care Training for Pediatric Hematology/Oncology Fellows. MedEdPORTAL. 2017;13:10543. 10.15766/mep_2374-8265.10543. 10.15766/mep_2374-8265.10543 PMC6342230 30800745

[98] Van Schaik Eileen, Howson Alex, Sabin Janice. Healthcare Disparities. MedEdPORTAL. 2014;10. 10.15766/mep_2374-8265.9675.

[99] Dovidio JF, Penner LA, Albrecht TL, Norton WE, Gaertner SL, Shelton JN. Disparities and distrust: The implications of psychological processes for understanding racial disparities in health and health care. Soc Sci Med. 2008;67(3):478-86. 10.1016/j.socscimed.2008.03.019. 10.1016/j.socscimed.2008.03.019 18508171

[100] Weight Bias in Health Care.; 2009. https://www.youtube.com/watch?v=lZLzHFgE0AQ&feature=player_embedded. Accessed 12 Jan 2021.

[101] Celik HH, Klinge II, Weijden TT van der, Widdershoven GGAM, Lagro-Janssen TALM. Gender sensitivity among general practitioners: results of a training programme. BMC Med Educ. 2008;8:36. 10.1186/1472-6920-8-36. 10.1186/1472-6920-8-36 PMC2446386 18582361

[102] Sukhera J, Watling CJ. A Framework for Integrating Implicit Bias Recognition Into Health Professions Education. Acad Med. 10.1097/ACM.0000000000001819 28658015

[103] Culhane-Pera KA, Reif C, Egli E, Baker NJ, Kassekert R. A curriculum for multicultural education in family medicine. Fam Med. 1997;29(10):719-23. 9397362

[104] Ballon BC, Skinner W. “Attitude is a Little Thing That Makes a Big Difference”: Reflection Techniques for Addiction Psychiatry Training. Acad Psychiatry. 2008;32(3):218-24. 10.1176/appi.ap.32.3.218. 10.1176/appi.ap.32.3.218 18467479

[105] Murray-Garcia JL, Harrell S, Garcia JA, Gizzi E, Simms-Mackey P. Self-reflection in multicultural training: be careful what you ask for. Acad Med. 2005;80(7):694-701. 10.1097/00001888-200507000-00016 15980089

[106] Wu S, Li L, Wu Z, et al. A brief HIV stigma reduction intervention for service providers in China. AIDS Patient Care STDs. 2008;22(6):513-20. 10.1089/apc.2007.0198. 10.1089/apc.2007.0198 PMC2700336 18462076

[107] Oanh K, Ashburn K, Pulerwitz J, Ogden J, Nyblade L. Improving hospital-based quality of care in Vietnam by reducing HIV-related stigma and discrimination. HIV AIDS. Published online 2008. 10.31899/HIV2.1013.

[108] Nyblade L, Stangl A, Weiss E, Ashburn K. Combating HIV stigma in health care settings: what works? J Int AIDS Soc. 2009;12:15. 10.1186/1758-2652-12-15. 10.1186/1758-2652-12-15 PMC2731724 19660113

[109] Mahendra V, Gilborn L, George B, et al. Reducing AIDS-related stigma and discrimination in Indian hospitals. HIV AIDS. 2006. 10.31899/HIV2.1027.

[110] Li L, Wu Z, Liang LJ, et al. Reducing HIV-related stigma in health care settings: a randomized controlled trial in China. Am J Public Health. 2013;103(2):286-92. 10.2105/AJPH.2012.300854. 10.2105/AJPH.2012.300854 PMC3556241 23237175

[111] Mutha S, Allen C, Welch M. Toward Culturally Competent Care: A Toolbox for Teaching Communication Strategies. Center for the Health Professions, University of California, San Francisco; 2002.

[112] Hollenbach A, Eckstrand K, Dreger A. Implementing Curricular and Institutional Climate Changes to Improve Health Care for Individuals Who are LGBT, Gender Nonconforming, or Born with DSD: A Resource for Medical Educators. Assoc Am Med Coll.

[113] Like RC, Steiner RP, Rubel AJ. STFM Core Curriculum Guidelines. Recommended core curriculum guidelines on culturally sensitive and competent health care. Fam Med. 1996;28(4):291-7. 8728526

[114] Garrison CB, McKinney-Whitson V, Johnston B, Munroe A. Race matters: Addressing racism as a health issue. Int J Psychiatry Med. 2018;53(5-6):436-44. 10.1177/0091217418791432. 10.1177/0091217418791432 30058464

[115] Hofmeister S, Soprych A. Teaching resident physicians the power of implicit bias and how it impacts patient care utilizing patients who have experienced incarceration as a model. Int J Psychiatry Med. 2017;52(4-6):345-54. 10.1177/0091217417738935. 10.1177/0091217417738935 29179660

[116] Zeidan A, Tiballi A, Woodward M, Di Bartolo IM. Targeting Implicit Bias in Medicine: Lessons from Art and Archaeology. West J Emerg Med. 2019;21(1):1-3. 10.5811/westjem.2019.9.44041. 10.5811/westjem.2019.9.44041 PMC6948688 31913809

[117] Poitevien P, Osman C. Tackling Implicit and Explicit Bias Through Objective Structured Teaching Exercises for Faculty. J Grad Med Educ. 2018;10(3):353-54. 10.4300/JGME-D-17-00906.1. 10.4300/JGME-D-17-00906.1 PMC6008013 29946404

[118] Maina I. A systematic review of implicit racial bias in healthcare. Pediatrics. 2018;141(1). 10.1542/peds.141.1-MeetingAbstract.337.

[119] Meltzer EC, Suppes A, Burns S, et al. Stigmatization of substance use disorders among internal medicine residents. Subst Abuse. 2013;34(4):356-62. 10.1080/08897077.2013.815143. 10.1080/08897077.2013.815143 24159906

