# Supplementary material for: Confronting implicit bias toward patients: a scoping review of post-graduate physician curricula
Source: BMC Med Educ. 2022 Sep 29;22:696. doi: 10.1186/s12909-022-03720-0 (PMC9520104; doi:10.1186/s12909-022-03720-0)
Supplement: Supplementary file 1 — Additional file 1: Appendix 1. Search strategy of post-graduate physician implicit bias curricula in MEDLINE (Ovid), Embase, Web of Science, ERIC, CINAHL, and PsycINFO in February 2020. [file 12909_2022_3720_MOESM1_ESM.docx]

**Appendix 1.** Search strategy of post-graduate physician implicit bias curricula in MEDLINE (Ovid), Embase, Web of Science, ERIC, CINAHL, and PsycINFO in February 2020.

MEDLINE (Ovid)

Ovid MEDLINE(R) and Epub Ahead of Print, In-Process & Other Non-Indexed Citations, Daily and Versions(R) 1946 to February 21, 2020

1775 Records

(exp prejudice/ OR exp social discrimination/ OR exp stereotyping/ OR ((implicit OR unconcious OR emotion* OR racial OR ethnic* OR black OR hispanic OR latin* OR asian OR obes* OR fat OR gender OR homosexual* OR sex OR sexual OR diabl* OR lgbt* OR lesbian* OR gay OR bisexual OR transgender* OR age OR elderly OR poverty OR poor OR social class OR socioeconomic OR economic status OR religio* OR faith OR christian* OR hindu* OR islam* OR muslim* OR jew* OR drug OR addict* OR subtance use* OR substance abuse* OR HIV OR disabled OR disability OR handicap* OR impairment OR impaired OR mentally ill OR mental illness*) adj6 (bias* or prejudice* or sterotyp*)).ab,ti)

AND

(exp education/ OR education.fs OR education*.ab,ti OR training*.ab,ti OR teach*.ab,ti OR instruction.ab,ti OR curricul*.ab,ti)

AND

(exp education, medical, graduate/ OR exp education, medical, continuing/ OR exp physicians/ OR graduate?.ab,ti OR postgrad*.ab,ti OR residen*.ab,ti OR fellow*.ab,ti OR house officer?.ab,ti OR intern?.ab,ti OR physician?.ab,ti OR surgeon?.ab,ti OR hospitalist?.ab,ti OR cme.ab,ti)

Embase (Elsevier)

1356 Records

('prejudice'/exp OR 'social discrimination'/exp OR 'stereotyping'/exp OR ((implicit OR unconcious OR emotion* OR racial OR ethnic* OR black OR hispanic OR latin* OR asian OR obes* OR fat OR gender OR homosexual* OR sex OR sexual OR diabl* OR lgbt* OR lesbian* OR gay OR bisexual OR transgender* OR age OR elderly OR poverty OR poor OR 'social class' OR socioeconomic OR 'economic status' OR religio* OR faith OR christian* OR hindu* OR islam* OR muslim* OR jew* OR drug OR addict* OR 'subtance use*' OR 'substance abuse*' OR HIV OR disabled OR disability OR handicap* OR impairment OR impaired OR 'mentally ill' OR 'mental illness*') NEAR/6 (bias* or prejudice* or sterotyp*)):ab,ti)

AND

('education'/exp OR education*:ab,ti OR training*:ab,ti OR teach*:ab,ti OR instruction:ab,ti OR curricul*:ab,ti)

AND

('graduate medical education'/exp OR 'postgraduate student'/exp OR 'resident'/exp OR 'continuing education'/exp OR 'physician'/exp OR graduate$:ab,ti OR postgrad*:ab,ti OR residen*:ab,ti OR fellow*:ab,ti OR 'house officer$':ab,ti OR intern$:ab,ti OR physician$:ab,ti OR surgeon$:ab,ti OR hospitalist$:ab,ti OR cme:ab,ti)

Web of Science (Clarivate Analytics)

Indexes=SCI-EXPANDED, SSCI, A&HCI, CPCI-S, CPCI-SSH, BKCI-S, BKCI-SSH, ESCI, CCR-EXPANDED, IC Timespan=All years

481 Records

TS=(("implicit" OR "unconcious" OR "emotion*" OR "racial" OR "ethnic*" OR "black" OR "hispanic" OR "latin*" OR "asian" OR "obes*" OR "fat" OR "gender" OR "homosexual*" OR "sex" OR "sexual" OR "diabl*" OR "lgbt*" OR "lesbian*" OR "gay" OR "bisexual" OR "transgender*" OR "age" OR "elderly" OR "poverty" OR "poor" OR "social class" OR "socioeconomic" OR "economic status" OR "religio*" OR "faith" OR "christian*" OR "hindu*" OR "islam*" OR "muslim*" OR "jew*" OR "drug" OR "addict*" OR "subtance use*" OR "substance abuse*" OR "HIV" OR "disabled" OR "disability" OR "handicap*" OR "impairment" OR "impaired" OR "mentally ill" OR "mental illness*") NEAR/6 ("bias*" OR "prejudice*" OR "sterotyp*"))

AND

TS=("education*" OR "training*" OR "teach*" OR "instruction" OR "curricul*")

AND

TS=("graduate$" OR "postgrad*" OR "residen*" OR "fellow*" OR "house officer$" OR "intern$" OR "physician$" OR "surgeon$" OR "hospitalist$" OR "cme")

ERIC (EBSCO Host)

64 Records

(DE ("Social Bias" OR "Gender Bias" OR "Racial Bias") OR TI (("implicit" OR "unconcious" OR "emotion*" OR "racial" OR "ethnic*" OR "black" OR "hispanic" OR "latin*" OR "asian" OR "obes*" OR "fat" OR "gender" OR "homosexual*" OR "sex" OR "sexual" OR "diabl*" OR "lgbt*" OR "lesbian*" OR "gay" OR "bisexual" OR "transgender*" OR "age" OR "elderly" OR "poverty" OR "poor" OR "social class" OR "socioeconomic" OR "economic status" OR "religio*" OR "faith" OR "christian*" OR "hindu*" OR "islam*" OR "muslim*" OR "jew*" OR "drug" OR "addict*" OR "subtance use*" OR "substance abuse*" OR "HIV" OR "disabled" OR "disability" OR "handicap*" OR "impairment" OR "impaired" OR "mentally ill" OR "mental illness*") N6 ("bias*" OR "prejudice*" OR "sterotyp*")) OR AB (("implicit" OR "unconcious" OR "emotion*" OR "racial" OR "ethnic*" OR "black" OR "hispanic" OR "latin*" OR "asian" OR "obes*" OR "fat" OR "gender" OR "homosexual*" OR "sex" OR "sexual" OR "diabl*" OR "lgbt*" OR "lesbian*" OR "gay" OR "bisexual" OR "transgender*" OR "age" OR "elderly" OR "poverty" OR "poor" OR "social class" OR "socioeconomic" OR "economic status" OR "religio*" OR "faith" OR "christian*" OR "hindu*" OR "islam*" OR "muslim*" OR "jew*" OR "drug" OR "addict*" OR "subtance use*" OR "substance abuse*" OR "HIV" OR "disabled" OR "disability" OR "handicap*" OR "impairment" OR "impaired" OR "mentally ill" OR "mental illness*") N6 ("bias*" OR "prejudice*" OR "sterotyp*")))

AND

(TI ("education*" OR "training*" OR "teach*" OR "instruction" OR "curricul*") OR AB ("education*" OR "training*" OR "teach*" OR "instruction" OR "curricul*"))

AND

(DE ("Graduate Medical Education" OR "Medical School Faculty") OR TI ((("graduate$" OR "postgrad*" OR "residen*" OR "fellow*" OR "house officer$" OR "intern$") AND ("medical" OR "medicine" OR "health")) OR "physician$" OR "surgeon$" OR "hospitalist$" OR "cme") OR AB ((("graduate$" OR "postgrad*" OR "residen*" OR "fellow*" OR "house officer$" OR "intern$") AND ("medical" OR "medicine" OR "health")) OR "physician$" OR "surgeon$" OR "hospitalist$" OR "cme"))

CINAHL Plus (EBSCO Host)

555 Records

(MH ("Prejudice" OR "Gender Bias" OR "Cultural Bias" OR "Discrimination+" OR "Stereotyping") OR TI (("implicit" OR "unconcious" OR "emotion*" OR "racial" OR "ethnic*" OR "black" OR "hispanic" OR "latin*" OR "asian" OR "obes*" OR "fat" OR "gender" OR "homosexual*" OR "sex" OR "sexual" OR "diabl*" OR "lgbt*" OR "lesbian*" OR "gay" OR "bisexual" OR "transgender*" OR "age" OR "elderly" OR "poverty" OR "poor" OR "social class" OR "socioeconomic" OR "economic status" OR "religio*" OR "faith" OR "christian*" OR "hindu*" OR "islam*" OR "muslim*" OR "jew*" OR "drug" OR "addict*" OR "subtance use*" OR "substance abuse*" OR "HIV" OR "disabled" OR "disability" OR "handicap*" OR "impairment" OR "impaired" OR "mentally ill" OR "mental illness*") N6 ("bias*" OR "prejudice*" OR "sterotyp*")) OR AB (("implicit" OR "unconcious" OR "emotion*" OR "racial" OR "ethnic*" OR "black" OR "hispanic" OR "latin*" OR "asian" OR "obes*" OR "fat" OR "gender" OR "homosexual*" OR "sex" OR "sexual" OR "diabl*" OR "lgbt*" OR "lesbian*" OR "gay" OR "bisexual" OR "transgender*" OR "age" OR "elderly" OR "poverty" OR "poor" OR "social class" OR "socioeconomic" OR "economic status" OR "religio*" OR "faith" OR "christian*" OR "hindu*" OR "islam*" OR "muslim*" OR "jew*" OR "drug" OR "addict*" OR "subtance use*" OR "substance abuse*" OR "HIV" OR "disabled" OR "disability" OR "handicap*" OR "impairment" OR "impaired" OR "mentally ill" OR "mental illness*") N6 ("bias*" OR "prejudice*" OR "sterotyp*")))

AND

(MH ("Education+") OR TI ("education*" OR "training*" OR "teach*" OR "instruction" OR "curricul*") OR AB ("education*" OR "training*" OR "teach*" OR "instruction" OR "curricul*"))

AND

(MH ("Education, Graduate" OR "Education, Medical, Continuing" OR "Internship and Residency" OR "Interns and Residents") OR TI ((("graduate$" OR "postgrad*" OR "residen*" OR "fellow*" OR "house officer$" OR "intern$") AND ("medical" OR "medicine" OR "health")) OR "physician$" OR "surgeon$" OR "hospitalist$" OR "cme") OR AB ((("graduate$" OR "postgrad*" OR "residen*" OR "fellow*" OR "house officer$" OR "intern$") AND ("medical" OR "medicine" OR "health")) OR "physician$" OR "surgeon$" OR "hospitalist$" OR "cme"))

PsycINFO (EBSCO Host)

447 Records

(DE ("Implicit Bias" OR "Prejudice" OR "Religious Prejudices" OR "Racism" OR "Ethnocentrism" OR "Sterotyped Attitudes" OR "Ageism" OR "Sexism" OR "Discrimination" OR "Social Discrimination" OR "Age Discrimination" OR "Disability Discrimination" OR "Sex Discrimination" OR "Disabled (Attitudes Toward)" OR "Homosexuality (Attitudes Toward)" OR "Transgender (Attitudes Toward)" OR "Obesity (Attitudes Toward)" OR "AIDS (Attitudes Toward)" OR "Aged (Attitudes Toward)") OR TI (("implicit" OR "unconcious" OR "emotion*" OR "racial" OR "ethnic*" OR "black" OR "hispanic" OR "latin*" OR "asian" OR "obes*" OR "fat" OR "gender" OR "homosexual*" OR "sex" OR "sexual" OR "diabl*" OR "lgbt*" OR "lesbian*" OR "gay" OR "bisexual" OR "transgender*" OR "age" OR "elderly" OR "poverty" OR "poor" OR "social class" OR "socioeconomic" OR "economic status" OR "religio*" OR "faith" OR "christian*" OR "hindu*" OR "islam*" OR "muslim*" OR "jew*" OR "drug" OR "addict*" OR "subtance use*" OR "substance abuse*" OR "HIV" OR "disabled" OR "disability" OR "handicap*" OR "impairment" OR "impaired" OR "mentally ill" OR "mental illness*") N6 ("bias*" OR "prejudice*" OR "sterotyp*")) OR AB (("implicit" OR "unconcious" OR "emotion*" OR "racial" OR "ethnic*" OR "black" OR "hispanic" OR "latin*" OR "asian" OR "obes*" OR "fat" OR "gender" OR "homosexual*" OR "sex" OR "sexual" OR "diabl*" OR "lgbt*" OR "lesbian*" OR "gay" OR "bisexual" OR "transgender*" OR "age" OR "elderly" OR "poverty" OR "poor" OR "social class" OR "socioeconomic" OR "economic status" OR "religio*" OR "faith" OR "christian*" OR "hindu*" OR "islam*" OR "muslim*" OR "jew*" OR "drug" OR "addict*" OR "subtance use*" OR "substance abuse*" OR "HIV" OR "disabled" OR "disability" OR "handicap*" OR "impairment" OR "impaired" OR "mentally ill" OR "mental illness*") N6 ("bias*" OR "prejudice*" OR "sterotyp*")))

AND

(DE ("Education" OR "Graduate Education" OR "Medical Education" OR "Continuing Education" OR "Medical Internship" OR "Medical Residency") OR TI ("education*" OR "training*" OR "teach*" OR "instruction" OR "curricul*") OR AB ("education*" OR "training*" OR "teach*" OR "instruction" OR "curricul*"))

AND

DE ("Physicians" OR "Family Physicians" OR "General Practitioners" OR "Gynecologists" OR "Internists" OR "Neurologists" OR "Obstetricians" OR "Pathologists" OR "Pediatricians" OR "Psychiatrists" OR "Surgeons" OR "Medical Internship" OR "Medical Residency") OR TI ((("graduate$" OR "postgrad*" OR "residen*" OR "fellow*" OR "house officer$" OR "intern$") AND ("medical" OR "medicine" OR "health")) OR "physician$" OR "surgeon$" OR "hospitalist$" OR "cme") OR AB ((("graduate$" OR "postgrad*" OR "residen*" OR "fellow*" OR "house officer$" OR "intern$") AND ("medical" OR "medicine" OR "health")) OR "physician$" OR "surgeon$" OR "hospitalist$" OR "cme"))
