# Supplementary material for: Confronting implicit bias toward patients: a scoping review of post-graduate physician curricula
Source: BMC Med Educ. 2022 Sep 29;22:696. doi: 10.1186/s12909-022-03720-0 (PMC9520104; doi:10.1186/s12909-022-03720-0)
Supplement: Supplementary file 2 — Additional file 2: Appendix2. Data extraction form for scoping review on curricula addressing post-graduate physician implicit bias toward patients. [file 12909_2022_3720_MOESM2_ESM.docx]

**Appendix 2.** Data extraction form for scoping review on curricula addressing post-graduate physician implicit bias toward patients.

**Basic study details**

Study ID

ID# assigned by Covidence

_____________________________________________________

Title

_____________________________________________________

First author, year of publication

_____________________________________________________

Journal

_____________________________________________________

Country in which study was conducted

_____________________________________________________

Institution from which study came

_____________________________________________________

Full paper or abstract?

1. Full paper
2. Abstract only

Description of curriculum implementation or recommendations for intervention?

1. Outlines curriculum which was implemented
2. Did not implement intervention but provides suggestions for curriculum

One-line summary of the paper

If it describes actual intervention, include type of bias, participants, and general educational approach.

If it describes suggestions for curriculum only, include type of bias and main recommendations.

_____________________________________________________

**Concept: purpose of intervention**

Educational theory

Theory, concept, approach, or any other process that led to design of intervention

_____________________________________________________

Type of bias addressed

What type of implicit bias? List multiple if necessary.

_____________________________________________________

Term used for implicit bias

Did they primarily use another term to refer to implicit bias (e.g. stigma, prejudice, racism)?

_____________________________________________________

Dedicated implicit bias curriculum vs integrated into larger curriculum?

Was the intervention intended to primarily address implicit bias? If not, what was the focus of the intervention?

_____________________________________________________

Learning objectives

Include all learning objectives, not just those specific to implicit bias.

_____________________________________________________

**Context: curriculum intervention**

Population of learners (level of training)

1. Resident and/or fellow physicians
2. Attending/practicing physicians
3. Both resident and attending physicians
4. Other

Population of learners (specialty)

_____________________________________________________

Number of learners (post-graduate physicians only)

_____________________________________________________

Other demographics of learners provided (post-graduate physicians only)

_____________________________________________________

Facilitators’ background

Professional discipline, teaching experience, and other details provided about instructors

_____________________________________________________

Facilitator preparation

How were facilitators prepared to conduct intervention?

_____________________________________________________

Learner: facilitator ratio

_____________________________________________________

Mode of intervention

Didactic lectures, clinical rotation, online modules, partnering with patient representatives, etc.

_____________________________________________________

Materials

Describe any materials provided to learns or used in the training of facilitators

_____________________________________________________

Content of curriculum

Specifics of what was taught and how it was delivered

_____________________________________________________

Environment

Where was intervention held (hospital ward, community, university lecture hall, etc.)?

_____________________________________________________

Length/frequency of curriculum

Details about schedule of intervention: how long were workshops, how frequently were they implemented?

_____________________________________________________

Reimbursements or incentives provided to learners

Residency graduation requirements, free meals, financial incentives, etc.

_____________________________________________________

Challenges encountered

What obstacles or challenges to curriculum intervention came up?

_____________________________________________________

Modifications to intervention

If the educational intervention was modified during its implementation, describe how and why

_____________________________________________________

**Outcomes/results**

Were outcomes of intervention measured?

1. Yes
2. No

Methods for measuring outcomes

Pre/post questionnaires, pre/post exam, pre/post IAT, etc. Include name of survey if applicable

_____________________________________________________

Outcomes of intervention

What were the outcomes of the intervention, statistical test used, and statistical significance?

_____________________________________________________

Attendance

Learner attendance and how this was assessed

_____________________________________________________

**Authors’ analysis of intervention**

Evidence for need for implicit bias curricula

What specific evidence did the results/analysis of this curriculum provide for the need for implicit bias interventions?

_____________________________________________________

Evidence for conceptual framework of curriculum

What specific evidence supports this particular curriculum? Focus on implicit bias part of curriculum

_____________________________________________________

Strengths of curriculum reported by authors

Specific to implicit bias curriculum

_____________________________________________________

Weaknesses of curriculum reported by authors

Specific to implicit bias curriculum

_____________________________________________________

Future directions

What implications does this curriculum have for the future of implicit bias interventions?

_____________________________________________________
