# Supplementary material for: Confronting implicit bias toward patients: a scoping review of post-graduate physician curricula
Source: BMC Med Educ. 2022 Sep 29;22:696. doi: 10.1186/s12909-022-03720-0 (PMC9520104; doi:10.1186/s12909-022-03720-0)
Supplement: Supplementary file 3 — Additional file 3: Appendix 3. Summary of all 90 studies included in the scoping review (February-November 2020) of post-graduate physician implicit bias curricula [52–119]. [file 12909_2022_3720_MOESM3_ESM.docx]

**Appendix 3.** Summary of all 90 studies included in the scoping review (February-November 2020) of post-graduate physician implicit bias curricula.

| **Title** | **First author, year of publication** | **Institution** | **Summary of study** |
| --- | --- | --- | --- |
| Understanding Race, Ethnicity, and Power: The Key to Efficacy in Clinical Practice^52^ | Pinderhughes, 1989 | - | Details teaching methods for helping clinicians recognize bias and power differentials in working with patients of different racial/ethnic backgrounds |
| The Weight of Pain: What Does a 10 on the Pain Scale Mean? An Innovative Use of Art in Medical Education to Enhance Pain Management^53^ | Marr, 2019 | University of Maryland School of Medicine | Utilizes art/museums and constructivist learning theory to create sessions where learners observe how personal experiences, communication, and tolerance for uncertainty shape emotions and responses to patients in pain |
| Reducing mental illness stigma in mental health professionals using a web-based approach^54^ | Bayar, 2009 | Istanbul University | Investigates whether an internet-based anti-stigma campaign designed to focus on key issues of stigmatization and discrimination would be useful in reducing the stigma towards mentally ill persons among mental health professionals |
| Recommendations for teaching about racial and ethnic disparities in health and health care^55^ | Smith, 2007 | Virginia Commonwealth University | Presents the Society of General Internal Medicine Health Disparities Task Force's guidelines for medical education on disparities in health and health care, including learning objectives, suggested content, methods for teaching, and a set of current resources |
| Recognizing racism in medicine: A student-organized and community-engaged health professionals conference^38^ | Adelekun, 2018 | Perelman School of Medicine, University of Pennsylvania | Evaluation and implementation of a student-led educational conference designed to train health professionals on the impact of racism in health care and provide tools to mitigate it |
| Pediatric residents and young handicapped children: Curriculum evaluation^56^ | Richardson, 1978 | Children's Hospital National Medical Center | Curriculum designed to improve the knowledge, skills, attitudes, and clinical judgement of pediatric residents in regards to young handicapped children and their families |
| Patrolling your blind spots: introspection and public catharsis in a medical school faculty development course to reduce unconscious bias in medicine^16^ | Hannah, 2013 | - | Describes semester-long faculty development course for physicians to reduce unconscious bias |
| A multidisciplinary and culturally appropriate model of care in cardiac outreach clinic improves indigenous patient continuity of care^57^ | Bryce 2012 | Princess Alexandra Hospital University of Queensland; Southern Cardiac Clinical Network; University of South Australia Adelaide | Structured curriculum which consists of focus groups with aboriginal health care workers to inform development of a multidisciplinary team model of care "aligned with cultural safety and competence concepts" |
| Medical residents reflect on their prejudices toward poverty: a photovoice training project^28^ | Loignon, 2014 | Université de Sherbrooke | Utilizes photovoice study with family medicine residents to uncover and examine prejudices and assumptions about poverty, to help them overcome the social distance between themselves and their socioeconomically disadvantaged patients |
| Uthscsa safe space: Becoming an ally to the lgbtq communities^58^ | Alonzo, 2014 | University of Texas Health Science Center at San Antonio | 90 min lecture workshop to raise cultural competency and professionalism of students, faculty, and staff toward LGBTQ population |
| Using Non-Feature Films to Teach Diversity, Cultural Competence, and the DSM-IV-TR Outline for Cultural Formulation^59^ | Lim, 2008 | University of California, Davis School of Medicine | Utilizes nonfeature films to simulate the discussion of racism in cultural competence education |
| Learner Reactions to Activities Exploring Racism as a Social Determinant of Health^60^ | Dennis, 2019 | OU-TU School of Community Medicine, University of Oklahoma | Describes responses of learners to learning sessions on racism as a social determinant of health (SDOH) which highlights structural, personally-mediated, and internalized racism |
| Immediate and follow-up effects of a brief disability curriculum on disability knowledge and attitudes of PM&R residents: A comparison group trial^61^ | Moroz, 2010 | New York University School of Medicine | Examines brief sensitivity training on people with disabilities for physical medicine and rehabilitation residents towards |
| Understanding Our Own Biases as Surgeons: A Departmental Effort^62^ | Rickert, 2020 | Massachusetts General Hospital | Utilizes implicit-association test to understand and address racial bias among faculty and residents in surgery department |
| Local patients, local stories: A Latino cultural competency training program for healthcare providers^63^ | Baig, 2014 | University of Illinois College of Medicine | Development and assessment of a training program to improve care for Latinos with diabetes which uses patient photographs, patient stories, information about local resources, and exercises to increase awareness of stereotyping in the healthcare setting |
| HIV stigma intervention in a low-HIV prevalence setting: a pilot study in an Egyptian healthcare facility^64^ | Lohiniva, 2016 | National AIDS Program Infection Control Unit at the Global Disease Detection and Response Program of the US Naval Medical Research Unit No.3 | Uses intervention and control hospitals to test stigma intervention related to patients with HIV |
| Unconscious Bias: Addressing the Hidden Impact on Surgical Education^65^ | Backhus, 2019 | Stanford University | Recommends strategies to mitigate negative influence of unconscious bias in cardiothoracic surgeons and trainees, including establishing a longitudinal curriculum that may utilize tools such as IAT |
| Helping medical learners recognise and manage unconscious bias toward certain patient groups^20^ | Teal, 2011 | Baylor College of Medicine | Reviews educational strategies for helping medical students and residents develop awareness of implicit bias and address bias toward patients |
| Healthcare disparities^66^ | Okubanjo, 2017 | Advocate Christ medical center, emergency medicine residency | Health disparities curriculum which includes lecture entitled implicit bias and cultural competency |
| Health care disparities education using the implicit association test^67^ | Siegelman, 2016 | Emory University | Racial bias intervention for residents which includes introductory lecture, IAT, vignettes about black and white patients with acute and chronic pain, and facilitated discussion |
| Two Tailored Provider Curricula Promoting Healthy Weight in Lesbian and Bisexual Women^32^ | Ingraham, 2016 | HealthRight 360 | Curricula to reduce weight bias and stigma in treating lesbian and bisexual women who are overweight or obese |
| From surviving to advising: Pairing people with lived experience of mental health and addiction issues as advisors to senior psychiatry residents^26^ | Agrawal, 2016 | University of Toronto | Novel course that pairs service users as advisors to senior psychiatry residents with the goals of improving residents’ understanding of recovery, reducing negative stereotypes about people in recovery, and empowering service users |
| Food addiction: An overlooked cause of persistent overweight and obesity^68^ | Forbes, 2013 | Saybrook University | Mixed method analysis of how targeted education about food addiction and physician weight bias can change physicians' attitudes about overweight and obese patients |
| Influence of contact with schizophrenia on implicit attitudes towards schizophrenia patients held by clinical residents^69^ | Omori, 2021 | Nippon Medical School | Assesses the impacts of renaming the Japanese term for schizophrenia and contact with patients with schizophrenia on psychiatry residents' attitudes toward schizophrenia |
| Improving physician self-efficacy and reducing provider bias: A family science approach to pediatric obesity treatment^70^ | Eagleton, 2017 | Oklahoma State University | Describes use of human and family science in training primary care physicians to reduce weight bias and stigma in the treatment of childhood obesity |
| Improving Emergency Health Care Workers' Knowledge, Competency, and Attitudes Toward Lesbian, Gay, Bisexual, and Transgender Patients Through Interdisciplinary Cultural Competency Training^71^ | Bristol, 2018 | Mercy Medical Center | Assesses impact of LGBT cultural competency training on a sample of ED nurses, nurse practitioners, unit secretaries, and physicians |
| Implicit Stigma Recognition and Management for Health Professionals^27^ | Sukhera, 2019 | Schulich School of Medicine and Dentistry | Workshop to address implicit stigma recognition and management for physicians and nurses |
| Implicit Bias Training in a Residency Program: Aiming for Enduring Effects^19^ | Sherman, 2021 | University of Minnesota | Workshop for family medicine residents and faculty on structural racism and skills for managing implicit bias |
| Implicit Bias Education and Emergency Medicine Training: Step One? Awareness^35^ | Zeidan, 2018 | University of Kentucky | Utilizes Implicit Association Test (IAT) on race followed by discussion on implicit bias to increase participant's awareness of individual implicit bias and how their bias affects patient care |
| Implicit bias affects us all: Simulation and panel discussion^72^ | Yang 2019 | - | Session that includes live simulation and panel discussion to examine unconscious bias toward patients |
| Implementing a self-developed cultural competency workshop in pediatric residency and assessing outcomes^73^ | Maksimowski, 2016 | Case Western Reserve University | Cultural competency workshop in pediatric residency utilizing didactic teaching, video-debriefing, small group sessions, and Objective Structured Clinical Encounters (OSCEs) |
| Impact of social determinants of health curriculum on resident empathy^74^ | Peralta, 2018 | Montefiore Medical Center/ Albert Einstein College of Medicine | Empathy-based learning curriculum to increase resident comfort with exploring patients' social determinants of health through sessions covering topics like implicit bias |
| Training physicians on issues of race and racism to improve health care equity^75^ | Nelson, 2015 | Children's Hospitals and Clinics of Minnesota | Training module on race, racism and health care for residents |
| Evaluating the teaching of gender-specific medicine in postgraduate training for general practitioners^76^ | Dielissen, 2009 | - | Program to sensitize general practice residents and trainers to gender awareness by utilizing lecture, group exercises, and video consults based on guidelines from the Dutch College of General Practitioners |
| Envisioning health: A trans-disciplinary, community engaged visual intervention for healthcare providers on implicit bias toward Latino/a immigrant youth^77^ | Lightfoot, 2015 | University of North Carolina | Allowed pediatric residents to learn how Latinx patients' life and health is influenced by Latino ethnicity and experience of migration by having Latinx adolescents share photos they created |
| The Efficacy of an Antioppression Curriculum for Health Professionals^78^ | Wu, 2019 | University of California San Francisco | Describes curriculum based on antioppression framework which encourages health professionals to evaluate their biases and combat health care disparities through active process of allyship |
| Education as a tool to modify anti-obesity bias among Pediatric residents^79^ | Rincon-Subtirelu, 2017 | Children's Mercy Hospital and Clinics | Obesity curriculum which utilizes IAT, lecture series, reading material, and video to assess and address pediatric residents' anti-obesity bias |
| Does awareness of unconscious associations enhance learning about healthcare disparities?^80^ | Sabin, 2010 | University of Washington | Brief presentation of interactive e-learning course designed for physicians and other providers to improve self-awareness of implicit bias |
| A decade of change in attitudes toward the homeless among primary care internal medicine residents^81^ | Norlock, 2014 | Stroger Hospital of Cook County | Two-week homeless medicine rotation for internal medicine residents |
| Culturally appropriate training to build better relationships between men who have sex with men (MSM) of color and their health providers^82^ | Cropper-Williams 2018 | HealthHIV | Continuing Medical Education (CME) course developed to improve relationships between providers and men who have sex with men (MSM) of color, with a specific focus on implicit bias |
| Cultural competence education in a simulated clinical environment: A pilot experience^21^ | Paroz 2014/2016 | University of Lausanne | Didactic videos, powerpoint presentations, written clinical cases, and 2 simulated patient encounters to train internal and general medicine resident physicians in cultural competence |
| Cultural and structural competency training for medical residents^83^ | Diaz Del Carpio 2018 | State University of New York at Buffalo; Jacobs School of Medicine and Biomedical Sciences | Describes workshops implemented with emergency medicine and medicine residents to address cultural competence, unconscious bias, stereotyping, microaggressions, and factors related to health disparities including poverty, lack of transportation, chronic illness, language, and culture |
| Cultivating compassionate care, advocacy skills and a health equity lens in resident physicians: The development of a social paediatrics curriculum^84^ | Ogilvie 2019 | Schulich School of Medicine and Dentistry, Western University | Longitudinal social pediatrics curriculum on health equity, vulnerable populations, and implicit bias |
| A council of elders: creating a multi-voiced dialogue in a community of care^85^ | Katz, 2000 | Cambridge Health Alliance Harvard Medical School | Describes collaboration of elders and primary care residents to identify ways to overcome geriatric health problems and address clinician ageism |
| Evaluation of a workshop intervention to reduce racial bias in internal medicine residents' clinical decision-making^86^ | Stahr 2017 | University of Wisconsin | Evaluation of the extent to which workshop intervention can reduce race bias in internal medicine residents’ clinical decision making |
| Evaluation of a Training to Reduce Provider Bias Toward Pregnant Patients With Substance Abuse^87^ | Seybold 2014 | Charleston Area Medical Center Health Education and Research Institute | Provides framework for training to increase providers' knowledge surrounding substance abuse treatment and decrease bias towards substance-abusing women, specifically pregnant women in rural communities |
| Comprehensive Internal Medicine Residency Curriculum on Primary Care of Patients Who Identify as LGBT^88^ | Ufomata, 2018 | University of Pittsburgh | Initiation of a case-based curriculum about LGBT health with component related to implicit bias |
| Am i biased? Using the implicit association test to start the conversation among internal medicine residents^89^ | Sanchez, 2018 | University of Pennsylvania | Brief presentation of implicit bias intervention using IAT with Internal Medicine trainees |
| Addressing racial bias in wards^90^ | Tsai, 2018 | Brown University | Discusses several evidence-based steps to guide discussions around race in clinical settings and mitigate bias and racism in the practice of medicine |
| Addressing Implicit Bias to Improve Cross-cultural Care^91^ | Pereda 2018 | University of New Mexico | Provides evidence for the importance of race-conscious training and briefly describes cultural humility program based on critical race theory |
| Caring for LGBTQ patients: Methods for improving physician cultural competence^92^ | Klein, 2016 | Providence Oregon Family Medicine Residency Program | Curriculum to teach family medicine residents and faculty about LGBTQ patients' needs for better health care |
| An academic half-day for healthcare disparities and social justice^93^ | Shutak 2017 | University of Minnesota | Describes academic half day focused on healthcare disparities, which addresses topics like stereotype threat through didactic lecture, small group work, and large group discussion |
| Early impact of a health equity, diversity, and inclusion curricula on resident knowledge, attitudes, and skill in cross-cultural care^94^ | Pryce, 2019 | Cohen Children's Medical Center | Brief description of Health Equity, Diversity, and Inclusion (HEDI) curriculum at pediatric medical center to address residents' implicit bias and other sources of inequity |
| The Clinical Assessment of Substance Use Disorders^95^ | Daetwyler, 2012 | Drexel University College of Medicine | Communication skills module on societal and health care provider stigma toward patients with substance use disorders, which uses written text and instructional videos to facilitate establishment of therapeutic relationships and to motivate patients for treatment |
| Using Multimedia in Faculty Development on Multicultural Education: Scenes From the Movie “Crash”^96^ | Lypson, 2010 | University of Michigan | Participants view specific scenes of the 2004 movie Crash and then engage in individual writing, small group discussion, and large group sessions to reflect on race, gender, sexual orientation and socioeconomic diversity |
| Cross-Cultural Care Training for Pediatric Hematology/Oncology Fellows^97^ | Rao, 2017 | Mayo Clinic | Describes implementation of cross-cultural care curriculum for pediatric hematology/oncology fellows to navigate patients' social and cultural belief systems |
| Healthcare Disparities^98^ | Van Schaik, 2014 | - | Course on topics like the patient-physician relationship's contribution to disparities and how unconscious bias may conflict with consciously-held egalitarian values |
| Health Equity Rounds: An Interdisciplinary Case Conference to Address Implicit Bias and Structural Racism for Faculty and Trainees^25^ | Perdomo, 2019 | Boston Medical Center Boston Combined Residency Program | Describes Health Equity Rounds (HER), a longitudinal case-based curriculum on implicit bias and racism for practitioners across training levels and disciplines |
| Educating Clinicians About Cultural Competence and Disparities in Health and Health Care^41^ | Like, 2011 | Robert Wood Johnson Medical School | Summarizes and critiques the current continuing medical education offerings on cultural competence/disparities |
| Disparities and distrust: The implications of psychological processes for understanding racial disparities in health and health care^99^ | Dovidio, 2008 | Yale University, Wayne State University, University of Connecticut, University of Delaware, and Princeton University | Explores bias toward people who are Black, the implications it has for racial disparities in health care, and how to address unconscious attitudes and beliefs through counter-stereotype characteristics, self-regulation, and redirecting ingroup bias |
| Weight Bias in Health Care^100^ | Leone, 2009 | Rudd Center for Food Policy and Obesity at Yale University | Video which presents prevalence of obesity, prevalence of weight bias, consequences for patients confronting weight bias, examples of weight bias in health care setting, and reflective questions clinicians can ask themselves to detect their own weight bias |
| Gender sensitivity among general practitioners: results of a training programme^101^ | Celik, 2008 | Maastricht University | Aims to investigate whether general practitioners' gender sensitivity can be stimulated by a training program |
| Recognizing Privilege and Bias: An Interactive Exercise to Expand Health Care Providers’ Personal Awareness^39^ | Holm, 2017 | Henry Fold Health System | Describes Privilege and Responsibility Curricular Exercise (PRCE) to enhance health care providers' awareness of privilege and bias and leverage their advantages to reduce health care inequities |
| Addressing Racism in Medical Education An Interactive Training Module^15^ | White-Davis, 2018 | Montefiore Medical Center-Albert Einstein College of Medicine | Workshop and toolkit to help medical educators teach and address racism, presented at the 2016 Society of Teachers of Family Medicine (STFM) Annual Spring Conference |
| Breaking the Silence: Time to Talk About Race and Racism^37^ | Acosta, 2017 | University of California Davis Health System | Recommendations for curriculum on race and racism for health professional students and faculty to engage in, sustain, and deepen interracial dialogue |
| A Framework for Integrating Implicit Bias Recognition Into Health Professions Education^102^ | Sukhera, 2018 | Schulich School of Medicine and Dentistry, Western University | Proposes six-point actionable framework for integrating implicit bias recognition and management into health professions education that draws on the work of previous researchers and includes practical tools to guide curriculum developers |
| How to Make or Break Implicit Bias Instruction: Implications for Curriculum Development^29^ | Gonzalez, 2018 | Albert Einstein College of Medicine | Identifies common challenges to teaching implicit bias via qualitative interviews with faculty experienced in teaching the subject |
| Structural competency: theorizing a new medical engagement with stigma and inequality^17^ | Metzl, 2014 | - | Proposes new model to replace cultural competency, termed "structural competency," as pedagogical approach to stigma and inequalities |
| A curriculum for multicultural education in family medicine^103^ | Culhane-Pera, 1997 | Regions Hospital, St. Paul Ramsey | Describes a formal educational program for family practice residents on the influence of culture in the medical setting |
| “Attitude is a Little Thing That Makes a Big Difference”: Reflection Techniques for Addiction Psychiatry Training^104^ | Ballon, 2008 | University of Toronto | Incorporates reflection techniques in addiction psychiatry core rotation in order to increase critical self-awareness of attitudes, values, and beliefs in working with people with substance use and other addictive disorders |
| Self-reflection in multicultural training: be careful what you ask for^105^ | Murray-Garcia, 2005 | University of California, Davis | Addresses equity in health care delivery by applying racial identity theory via self-reflection with a focus on white physician trainees |
| A brief HIV stigma reduction intervention for service providers in China^106^ | Wu, 2008 | - | Incorporated role-plays, group discussions, games, HIV advocate testimony, and presentation from physicians specialized in AIDS care to reduce HIV stigma among physicians, nurses, and lab technicians in China |
| Improving hospital-based quality of care in Vietnam by reducing HIV-related stigma and discrimination^107^ | Oanh, 2008 | Institute for Social and Development Studies (ISDS) International Center for Research on Women (ICRW) Horizons Program | Compares 2 interventions to improve quality of care for patients with HIV in Vietnam, one focusing on reducing fear-based stigma and the other on reducing both fear-based and social stigma |
| Combating HIV stigma in health care settings: what works?^108^ | Nyblade, 2009 | International Center for Research on Women | Provides guidance on the importance of combatting HIV-related stigma and how to successfully address its causes and consequences in health care |
| Reducing AIDS-related stigma and discrimination in Indian hospitals^109^ | Mahendra, 2006 | Horizons Program Sharan Institute of Economic Growth | Identifies strengths and limitations of existing services for HIV infected individuals in hospitals, and uses this data to design educational interventions to make hospitals in New Delhi more “PLHA (People Living with HIV/AIDS)-friendly" |
| Reducing HIV-related stigma in health care settings: a randomized controlled trial in China^110^ | Li, 2013 | University of California at Los Angeles | Evaluates the results of intervention to reduce stigmatizing attitudes and behaviors towards people with HIV in China |
| Physicians and implicit bias: how doctors may unwittingly perpetuate health care disparities^18^ | Chapman, 2013 | University of Wisconsin-Madison | Suggests that the contribution of implicit bias to health care disparities could be reduced if all physicians acknowledged their susceptibility to such bias and deliberately practiced perspective-taking and individuation |
| Toward Culturally Competent Care: A Toolbox for Teaching Communication Strategies^111^ | Mutha, 2002 | UCSF | Detailed outline of cultural competency curriculum for health professionals which includes implicit bias exercises |
| Implementing Curricular and Institutional Climate Changes to Improve Health Care for Individuals Who are LGBT, Gender Nonconforming, or Born with DSD: A Resource for Medical Educators^112^ | Hollenbach, 2014 | American Association of Medical Colleges | Advisory committee on sexual orientation, gender identity, and sex development assembled by the AAMC which makes recommendations about how medical education can be more responsive to the health needs and outcomes of patients who are LGBT, gender nonconforming, etc. |
| Seeing Patients: Unconscious Bias in Health Care^34^ | White, 2011 | Harvard Medical School | Describes development of Culturally Competent Care Committee to institutionalize culturally competent care and provides practical suggestions for physicians to address bias |
| Mental illness-related stigma in healthcare: Barriers to access and care and evidence-based solutions^24^ | Knaak, 2017 | Mental Health Commission of Canada | Summarizes recommendations based on research in Canada for combatting stigma toward mental illness in healthcare settings |
| STFM Core Curriculum Guidelines. Recommended core curriculum guidelines on culturally sensitive and competent health care^113^ | Like, 1996 | Robert Wood Johnson Medical School, Department of Family Medicine | Provides extensive list of attitudes, knowledge, and skills that should be developed through medical education curricula focusing on culturally sensitive and competent health care |
| Race matters: Addressing racism as a health issue^114^ | Garrison, 2018 | Medical College of Wisconsin | Introduces program addressing racism as a health issue through integrated seminars for family medicine residents |
| Teaching resident physicians the power of implicit bias and how it impacts patient care utilizing patients who have experienced incarceration as a model^115^ | Hofmeister, 2017 | - | Describes method for "educating the educators" so that attending physicians can help residents address bias and increase self-reflection when interacting with patients who have experienced incarceration |
| Targeting Implicit Bias in Medicine: Lessons from Art and Archaeology^116^ | Zeidan, 2019 | University of Pennsylvania | Trainees were introduced to implicit bias through curriculum that focused on differentiating between objective and subjective assessments of historical objects in a museum |
| Tackling Implicit and Explicit Bias Through Objective Structured Teaching Exercises for Faculty^117^ | Poitevien, 2018 | NYU/Bellevue Hospital Pediatric Residency Program | Brief description of Objective Structured Teaching Exercises (OSTE) as tool for pediatric residents, fellows, and faculty to practice addressing implicit bias with standardized learners (SL) |
| A systematic review of implicit racial bias in healthcare^118^ | Maina, 2018 | - | Synthesizes the current knowledge on the role of implicit bias in healthcare disparities as well as interventions to address this and their outcomes |
| A systematic approach to faculty development in Women's health: lessons from education, feminism, and conflict theory^36^ | Neely, 2000 | MCP Hanemann School of Medicine | Targets faculty development in women's health through program with discussions and workshops on improving women's health education and outcomes |
| Stigmatization of substance use disorders among internal medicine residents^119^ | Meltzer, 2021 | Weill Cornell Medical College | Examines stigma toward substance use disorders (SUD) and assesses efficacy of an addiction medicine course in improving internal medicine residents' attitudes towards patients with SUD |
| Promoting skill-building in cultural competence: A must for paediatricians who care for socially vulnerable populations^22^ | Razack 2007 | McGill University | Provides specific recommendations for training pediatricians in cultural competence, with special attention to indigenous populations and immigrants |
| *Abbreviations: LGBTQ indicates lesbian, gay, bisexual, transgender, and queer*  *IAT indicates Implicit Association Test*  *- indicates not reported* | | | |
|  | | | |
